# Current Evidence on the Management of Non-occlusive Mesenteric Ischemia: A Comprehensive Narrative Review

**DOI:** 10.7759/cureus.114102

**Published:** 2026-08-07

**Authors:** Charis G Kourgiali, Kalliopi-Kleio E Gotti, Maria D Velikoudi, Dimitrios A Chatzelas

**Affiliations:** 1 2nd Department of Surgery, “G. Gennimatas” General Hospital, Faculty of Medicine, Aristotle University of Thessaloniki, Thessaloniki, GRC

**Keywords:** acute mesenteric ischemia, catheter-directed therapy, critical care, mesenteric perfusion, non-occlusive mesenteric ischemia

## Abstract

Non-occlusive mesenteric ischemia (NOMI) is a life-threatening form of acute mesenteric ischemia (AMI), caused by severe intestinal hypoperfusion in the absence of an obstructive lesion of the mesenteric arteries. Early diagnosis and management remain challenging because the condition often develops in critically ill patients and frequently presents with non-specific clinical manifestations. This comprehensive narrative review summarizes the current evidence regarding the management of NOMI. A comprehensive literature search was performed. Evidence relating to hemodynamic resuscitation, imaging, catheter-directed therapies, surgical management, bowel viability assessment, nutritional support, and postoperative care was critically synthesized. Current evidence supports immediate correction of the underlying low-flow state, optimization of systemic perfusion, and urgent multiphase computed tomography angiography as the cornerstones of management. Catheter-directed intra-arterial vasodilator therapy with papaverine may improve mesenteric perfusion in carefully selected patients without established bowel infarction, although supporting evidence remains predominantly observational. Conversely, patients with peritonitis, perforation, or convincing evidence of transmural necrosis require prompt surgical exploration, using damage-control principles when appropriate. Despite recent advances in critical care, imaging, and endovascular techniques, the available evidence remains limited by small retrospective studies and considerable heterogeneity. Early recognition, multidisciplinary management, and standardized institutional treatment pathways are likely to represent the most effective strategies for improving outcomes, while prospective multicenter studies are needed to establish evidence-based management algorithms and optimize patient selection for emerging therapeutic interventions.

## Introduction and background

Non-occlusive mesenteric ischemia (NOMI) is characterized by intestinal ischemia occurring despite the absence of an acute obstructive thrombus or embolus in the major mesenteric vessels [1]. The disorder was initially described in patients with advanced heart failure, and was traditionally attributed to intense mesenteric vasoconstriction during a systemic low-flow state [1,2]. Contemporary understanding is broader; NOMI represents a dynamic mismatch between intestinal oxygen delivery and metabolic demand, generated by varying combinations of reduced cardiac output, hypovolemia, systemic vasodilation, microcirculatory dysfunction, mesenteric vasoconstriction, venous congestion, increased intra-abdominal pressure, endothelial injury, and impaired oxygen extraction [3-6].

NOMI accounts for approximately 13-15% of acute mesenteric ischemia (AMI) in contemporary cohorts, although historical estimates have ranged more widely, because diagnostic definitions and case ascertainment differed substantially [7-9]. Population-based incidence has been estimated at approximately 0.9-2.0 cases per 100,000 persons per year [8,10]. Its clinical importance is disproportionate to its frequency. A systematic review estimated mortality at approximately 58%, while the prospective international Acute MESenteric Ischaemia study reported in-hospital and 90-day mortality of 72.7% and 74.5%, respectively [7,9]. The European Society for Vascular Surgery (ESVS) guidelines similarly identify NOMI as one of the most lethal forms of AMI [11].

Outcomes remain poor because NOMI usually develops in patients who are already critically ill. Sedation, mechanical ventilation, postoperative analgesia, altered consciousness, and competing organ dysfunction can conceal abdominal symptoms [3,5,6]. Furthermore, NOMI is not a binary vascular event but a continuum; reversible mucosal hypoperfusion may progress to full-thickness necrosis, while the proximal superior mesenteric artery (SMA) remains patent. Consequently, management must address both the underlying disorder and the threatened intestine [3-6].

The objective of this comprehensive narrative review is to critically evaluate and synthesize the current evidence on NOMI management, with particular emphasis on hemodynamic resuscitation, vasopressor strategy, intra-arterial vasodilator therapy, surgical decision-making, bowel-viability assessment, and post-treatment care. The review also aims to clarify the role of multidisciplinary treatment pathways and propose a practical management algorithm, while identifying major limitations in the available literature and priorities for future research.

## Review

Methods

A narrative literature review was performed using the PubMed/Medical Literature Analysis and Retrieval System Online (MEDLINE), Scopus, Web of Science, and Cochrane electronic databases up to June 2026. Search terms included: “non-occlusive mesenteric ischemia”, “nonocclusive mesenteric ischaemia”, “acute mesenteric ischemia”, “mesenteric vasoconstriction”, “low-flow mesenteric ischemia”, “papaverine”, “prostaglandin”, “intra-arterial vasodilator”, “catheter-directed therapy”, “computed tomography angiography”, “bowel viability”, “second-look laparotomy”, “cardiac surgery”, “hemodialysis”, and “abdominal compartment syndrome” in various combinations. The reference lists of relevant articles were screened for additional sources. Only articles published in English and indexed in peer-reviewed journals were included. Priority was given to systematic reviews, multicenter studies, comparative series, prospective observational cohorts, and larger case series. Due to heterogeneity in diagnostic definitions, interventions, follow-up, and outcome reporting, no quantitative analysis was performed.

The electronic search identified a large number of potentially relevant publications. Following the removal of duplicate records and screening for relevance, full-text articles addressing the management of NOMI were reviewed. Given the narrative nature of the review, no formal study selection process was undertaken. The final qualitative synthesis was based on the principal contemporary clinical practice guidelines, systematic reviews, observational studies, and landmark publications, covering pathophysiology, diagnosis, hemodynamic management, catheter-directed therapy, operative management, bowel viability assessment, and postoperative care.

The principal publications supporting each of these domains are cited within the corresponding sections of the review. Pathophysiology and clinical presentation were interpreted using landmark reviews and contemporary observational studies [1-18]; diagnostic evaluation was synthesized from imaging studies and international guidelines [6,16-24]; hemodynamic resuscitation and vasopressor management were based primarily on contemporary critical care recommendations and NOMI-specific studies [3-6,11-14]; catheter-directed therapy was interpreted from one systematic review, observational studies, and the current ESVS guidelines [25-31]; whereas operative management, bowel viability assessment, and postoperative care were synthesized from current guidelines and the principal surgical literature [6,24,29-40].

Pathophysiological basis of NOMI

The splanchnic circulation receives a substantial proportion of cardiac output and possesses strong autoregulatory capacity. During hypotension or reduced effective circulating volume, sympathetic activation and the renin-angiotensin-aldosterone and vasopressin systems redistribute blood toward the heart and brain. Mesenteric arterial constriction initially preserves systemic pressure but eventually reduces intestinal oxygen delivery [3,4]. Mesenteric vasoconstriction may persist even after macrocirculatory variables have apparently normalized. Reperfusion can then amplify epithelial injury through oxidative stress, neutrophil activation, endothelial dysfunction, capillary leakage, and disruption of the mucosal barrier. Bacterial translocation and release of inflammatory mediators may aggravate the systemic shock, creating a self-perpetuating cycle of intestinal ischemia, inflammation, vasopressor dependence, and multi-organ failure [3,5].

The intestinal lesions are frequently segmental and discontinuous. The mucosa and villous tips are affected first because of their high metabolic requirements and countercurrent oxygen exchange. Progressive injury extends into the submucosa and muscularis and may ultimately cause transmural necrosis, perforation, and peritonitis [3,5,6]. This heterogeneity explains why apparently viable and necrotic segments can alternate and why a single intra-operative inspection may underestimate evolving disease.

Clinical settings and recognition of patients at-risk

Typical precipitating conditions include cardiogenic shock, severe heart failure, hemorrhage, dehydration, septic shock, major burns, cardiac arrest, cardiac or aortic surgery, dialysis-associated hypotension, vasoactive drug exposure, and intra-abdominal hypertension (IAH). Digitalis and sympathomimetic or vasoconstrictive drugs have historically been implicated, while cocaine and other recreational stimulants may produce intense mesenteric vasoconstriction [2,4-6].

Cardiac surgery is one of the best-characterized settings. Advanced age, renal insufficiency, preoperative diuretic treatment, low cardiac output, prolonged cardiopulmonary bypass, hypothermia, intra-aortic balloon-pump support, high vasopressor requirements, and postoperative lactate elevation have been associated with NOMI [12-15]. In a Japanese database of 221,900 cardiac and aortic procedures, NOMI was recorded in 124 patients, representing only 0.06% of the cohort, but the in-hospital mortality reached 77% [16]. The low recorded incidence probably reflects both true rarity and underdiagnosis.

Patients receiving chronic hemodialysis are susceptible because of diffuse vascular disease, repeated hypotension, reduced circulatory reserve, and aggressive ultrafiltration. NOMI should be considered when new abdominal pain, distension, gastrointestinal bleeding, unexplained acidosis, or hemodynamic deterioration occurs during or shortly after dialysis [5,6]. After ruptured abdominal aortic aneurysm repair, several mechanisms may co-exist: hemorrhagic shock, vasopressor exposure, loss of inferior mesenteric artery perfusion, micro-embolization, reperfusion injury, and abdominal compartment syndrome (ACS). Although ischemic colitis after aortic surgery may have a partly distinct distribution and pathogenesis, severe non-occlusive hypoperfusion can involve both the small bowel and colon [6,17].

In the conscious patient, abdominal pain out of proportion to physical findings remains an important warning. In critically ill patients, more subtle findings predominate: increasing vasopressor requirement, feeding intolerance, abdominal distension, ileus, gastric retention, unexplained diarrhea, gastrointestinal bleeding, rising lactate, metabolic acidosis, or progressive organ failure. Peritoneal signs usually indicate advanced transmural injury and should not be awaited before investigation [5,6,11].

Diagnostic evaluation

Laboratory Findings

No laboratory marker can independently confirm or exclude NOMI. Lactate elevation reflects systemic hypoperfusion, impaired clearance, adrenergic stimulation, or established intestinal injury, and may remain normal during early or localized ischemia. Therefore, a single normal lactate concentration should not delay imaging [18,19]. Serial trends are more clinically useful than an isolated value, particularly when interpreted alongside vasopressor requirements, acid-base status, organ function, and abdominal findings [18].

Leukocytosis, elevated C-reactive protein, metabolic acidosis, base deficit, hyperkalemia, increased lactate dehydrogenase, creatine kinase, D-dimer, and transaminase elevation are non-specific. Intestinal fatty-acid binding protein, citrulline, D-lactate, smooth-muscle protein, ischemia-modified albumin, and endothelin-1 have been investigated, but none has sufficient validation for routine decision-making [18-21]. In a cardiac surgery cohort, postoperative endothelin-1 showed an association with NOMI, but limited sensitivity prevents its use as a stand-alone screening test [20].

Computed Tomography Angiography

Multiphase computed tomography angiography (CTA) is currently the first-line imaging examination when NOMI is suspected [11]. It is rapidly available, evaluates the bowel and mesenteric vessels simultaneously, and identifies alternative causes of deterioration. The examination should ideally include thin-section arterial and venous phases; a non-contrast phase may help identify hemorrhage, vascular calcification, or hyperattenuating bowel contents [6,22-24].

Possible vascular findings include narrowing or irregularity of the SMA and its branches, reduced opacification, distal branch pruning, and diminished mesenteric venous filling. Bowel findings include reduced or absent mural enhancement, abnormal hyperenhancement following reperfusion, wall thickening or thinning, luminal dilatation, mesenteric edema, ascites, pneumatosis intestinalis, portal venous gas, and pneumoperitoneum [22-25]. A reduction in SMA diameter relative to previous imaging may support the diagnosis, but absolute diameter measurements are inconsistent and should not be used in isolation [23,24].

The absence of proximal occlusion is essential but not sufficient [11]. Critically ill patients may have chronic asymptomatic mesenteric stenosis combined with a superimposed low-flow state [6]. Conversely, pneumatosis or portal venous gas does not invariably indicate irreversible infarction; interpretation depends on bowel enhancement, distribution, clinical context, acidosis, and peritoneal findings [22-25].

Catheter Angiography

Digital-subtraction angiography (DSA) was historically considered the diagnostic gold standard [11]. Characteristic findings include SMA narrowing, spasm of branch origins, irregular "beading," poor filling of intramural vessels, and delayed venous return [2,26]. Its contemporary role is more selective because CTA is faster, less invasive, and evaluates extra-arterial pathology. DSA remains valuable when clinical suspicion is high, but CTA findings are equivocal, when mesenteric vasospasm must be distinguished from fixed disease, or when intra-arterial vasodilator treatment is planned [6,26]. Diagnostic and therapeutic angiography can be performed during the same procedure, reducing delay in selected patients [26-28].

Immediate resuscitation and reversal of the precipitating cause

NOMI treatment begins before definitive diagnostic confirmation when clinical suspicion is substantial [11]. The principal objective is to restore intestinal oxygen delivery while avoiding interventions that further compromise the mesenteric microcirculation [3,5,6]. Airway support, supplemental oxygen, mechanical ventilation when indicated, vascular access, continuous hemodynamic monitoring, and repeated assessment of lactate, acid-base balance, urine output, and organ function are required [6,11]. Crystalloid resuscitation should correct hypovolemia, but indiscriminate fluid loading can worsen bowel-wall edema, venous congestion, cardiac failure, and intra-abdominal pressure. Dynamic assessment of fluid responsiveness is preferable to fixed-volume administration [5,6,11].

Cardiac output should be optimized. In patients with predominant pump failure, inotropic support may be necessary. Dobutamine or milrinone can improve forward flow in selected patients, although hypotension and arrhythmia may limit their use [5,6,11]. Mechanical circulatory support may be appropriate in refractory cardiogenic shock, but intra-aortic balloon pumping and other devices do not eliminate the risk of intestinal hypoperfusion [11,12,14].

Sepsis requires rapid source identification, appropriate cultures, antimicrobial treatment, and source control. Hypoxemia, anemia, arrhythmia, hypothermia, electrolyte abnormalities, and excessive positive end-expiratory pressure should be corrected when they impair oxygen delivery or venous return [6,11,29].

Vasopressor strategy

Vasopressors are frequently unavoidable in shock and should not be withheld when required to maintain vital-organ perfusion. The practical goal is the lowest effective dose that provides adequate systemic perfusion, while the underlying cause is treated. Persistent escalation despite apparently adequate volume status should prompt reassessment for uncontrolled infection, cardiac dysfunction, bleeding, obstruction, or ACS [5,6,11].

Norepinephrine remains the standard first-line vasopressor in most forms of distributive shock, but all potent vasoconstrictors may adversely affect the splanchnic circulation. Vasopressin can reduce norepinephrine requirements, although its effects on mesenteric perfusion are context-dependent [3,5,6]. There is insufficient clinical evidence to establish a universally mesenteric-sparing vasopressor. The presence of NOMI should therefore lead to hemodynamic individualization, rather than abrupt withdrawal of life-sustaining vasoactive support [5,6,11]. Medications that can intensify mesenteric vasoconstriction should be discontinued when feasible. These may include digitalis, high-dose sympathomimetics, ergot derivatives, and other vasoconstrictive agents [2,5,6].

Antimicrobial prophylaxis and anticoagulation

Loss of mucosal integrity permits bacterial translocation and may transform regional ischemia into systemic sepsis. Broad-spectrum antibiotics covering enteric gram-negative and anaerobic organisms are commonly recommended, particularly when bowel necrosis is suspected, or the patient has shock or organ failure [6,29]. Direct clinical evidence specific to NOMI is limited, and regimens should follow local infection protocols.

Therapeutic anticoagulation is not a primary treatment for pure vasospastic NOMI, because no obstructing thrombus is present. Nevertheless, unfractionated heparin is sometimes administered during catheter-directed treatment to reduce catheter-related thrombosis and possible microthrombus formation. Its use should be individualized according to bleeding risk, recent surgery, coagulopathy, and whether an occult thrombotic component is suspected [6,11].

Catheter-directed intra-arterial vasodilator therapy

Selective infusion of a vasodilator into the SMA is the principal NOMI-specific endovascular treatment. It is intended to reverse mesenteric vasoconstriction, improve microvascular flow, and interrupt progression from reversible ischemia to infarction [6,11]. Papaverine is the most frequently reported agent. A commonly described protocol consists of an intra-arterial bolus of approximately 30-80 mg, followed by continuous infusion at 30-60 mg/hour for 24-72 hours, with the catheter positioned in the SMA [2,26,28,30]. Exact dosing varies among institutions. Repeat angiography or assessment of clinical, biochemical, and imaging responses may guide continuation.

Prostaglandin E1, nitroglycerin, tolazoline, and glucagon have also been used. No adequately powered comparative trial has established the optimal drug, dosage, infusion duration, or monitoring strategy. Papaverine can cause systemic hypotension, arrhythmia, and hepatic toxicity and may precipitate catheter complications. Prostaglandins may also cause hypotension and bleeding-related concerns [27,28,30]. A systematic review and meta-analysis involving 12 retrospective studies and 245 patients treated predominantly with papaverine or prostaglandin reported a pooled mortality of 40.3%. In four comparative studies, local vasodilator therapy was associated with lower mortality than standard treatment, but diagnostic criteria, case selection, treatment timing, and co-interventions varied substantially. The authors emphasized that the overall evidence was of low quality [27].

Winzer et al. retrospectively compared intra-arterial papaverine with conservative treatment. Thirty-day mortality was 65.7% in the intervention group and 96.8% with conservative management. Severe hyperlactatemia, acidosis, and high catecholamine requirements predicted poor outcomes [28]. Although the difference was striking, non-randomized allocation and potential differences in disease severity or treatment eligibility limit causal interpretation. In a prospective observational study of patients with severe shock, intra-arterial prostaglandin E1 produced greater lactate reduction than standard care, although overall mortality remained high. Patients whose lactate decreased substantially had better outcomes than non-responders, suggesting that early physiological response may help identify whether perfusion remains reversible [30].

The best candidates appear to be patients with early or potentially reversible NOMI, persistent clinical concern despite resuscitation, angiographic vasoconstriction, and no clear evidence of perforation or transmural necrosis [11,27,28,30]. Treatment should not delay laparotomy in a patient with peritonitis, perforation, or convincing evidence of irreversible bowel injury [6,11]. Catheter therapy and surgery are not necessarily competing strategies; vasodilator infusion can be used before, during, or after surgery when residual mesenteric vasospasm threatens the remaining bowel [27,28,30]. The ESVS guidelines state that intra-arterial papaverine or prostaglandin infusion under protective heparinization may be considered, assigning a class IIb recommendation, based on level C evidence [11]. This accurately reflects the present uncertainty; the treatment is physiologically compelling and supported by observational studies, but definitive comparative efficacy remains unproven.

Abdominal compartment syndrome

IAH impairs intestinal perfusion by reducing the arterial-intraluminal pressure gradient, compressing mesenteric veins and capillaries, increasing bowel-wall edema, and reducing cardiac output. IAH is defined by intra-abdominal pressure exceeding 12 mmHg, while ACS generally denotes sustained pressure above 20 mmHg accompanied by new organ dysfunction [31]. Bladder-pressure measurement should be performed in at-risk patients, including those with massive resuscitation, severe pancreatitis, abdominal trauma, ruptured aneurysm repair, major burns, tense abdominal distension, or otherwise unexplained respiratory, renal, and circulatory deterioration [11,31].

Initial measures include evacuation of intraluminal contents, drainage of intra-abdominal fluid when appropriate, optimization of analgesia and neuromuscular relaxation, avoidance of excessive fluid accumulation, and improvement of abdominal-wall compliance. Established ACS with organ dysfunction requires urgent decompressive laparotomy [31,32]. A meta-analysis found that decompression improves intra-abdominal pressure, oxygenation, and urine output, but mortality remained close to 50%, reflecting advanced underlying illness [32].

Indications for operative management

Immediate surgical exploration is indicated when there is generalized peritonitis, perforation, free intraperitoneal air, uncontrolled abdominal sepsis, or strong clinical and radiological evidence of transmural necrosis. Progressive shock or acidosis despite appropriate resuscitation may also justify exploration when the intestine remains a plausible source [6,11].

The objective is to remove irreversibly necrotic bowel while preserving every segment likely to recover [6,11]. NOMI frequently produces patchy injury, making resection margins difficult to define [3,5]. Clearly, gangrenous or perforated bowel must be removed, but extensive resection of borderline segments at the initial operation can produce avoidable short-bowel syndrome. Damage-control principles are often appropriate. In unstable patients, resection may be performed without immediate anastomosis, leaving the bowel ends stapled or exteriorized. Temporary abdominal closure facilitates ongoing resuscitation and planned reassessment. Restoration of continuity can be deferred until physiology improves and bowel viability is clearer [6,11].

A planned second-look operation, usually within 24-48 hours, is particularly valuable when large areas are marginal, vasopressor requirements remain high, or progressive ischemia is possible. At re-exploration, recovered bowel can be preserved, and newly demarcated necrotic segments resected. The interval should be shortened in case of clinical or laboratory deterioration [6,11].

Assessment of bowel viability

Traditional intraoperative bowel assessment relies on color, peristalsis, mesenteric pulsation, and bleeding from cut edges [6,11,33]. These findings are subjective and can be misleading during shock, hypothermia, or vasopressor therapy [3,5,6]. Palpable proximal arterial pulses do not exclude microcirculatory failure [3,33]. Handheld Doppler ultrasound may confirm flow in mesenteric or mural vessels but provides limited information about tissue oxygenation [6,33]. Fluorescein assessment, laser Doppler flowmetry, visible-light spectroscopy, and other perfusion-monitoring systems have been investigated but are not universally available [33-36].

Indocyanine-green fluorescence angiography provides real-time visualization of tissue perfusion and may alter resection margins or help preserve bowel that appears uncertain under white light [33]. However, fluorescence intensity is affected by dose, timing, cardiac output, edema, camera distance, and ambient conditions. Most evidence comes from small observational studies, and validated thresholds separating reversible from irreversible ischemia are lacking [33-36]. Therefore, it should supplement, not replace, surgical judgment and second-look evaluation.

Diagnostic laparoscopy may be useful in selected stable patients or when transport to radiology is hazardous. Its limitations include incomplete bowel assessment, difficulty evaluating mucosal injury, and the possibility that early ischemia appears deceptively mild. Open exploration remains preferable when extensive necrosis, severe distension, ACS, or major resection is anticipated [6,11,31].

Postoperative and supportive care

Successful bowel resection does not correct the mechanism responsible for NOMI. Hemodynamic optimization, treatment of sepsis or cardiac dysfunction, minimization of unnecessary vasoconstrictors, and surveillance for recurrent ischemia ought to continue. Persistent lactate elevation, acidosis, increasing vasopressor requirements, abdominal distension, or new organ failure should prompt urgent reassessment. Fluid balance is important. Both recurrent hypovolemia and progressive fluid overload can impair intestinal perfusion [5,6,11]. Electrolytes, glucose, temperature, hemoglobin, coagulation, renal function, and acid-base status should be managed actively. Enteral nutrition should be withheld during overt or uncontrolled intestinal ischemia [6,11,37].

Feeding increases intestinal metabolic demand and may exacerbate the oxygen supply-demand mismatch when perfusion remains inadequate [6,37]. Once hemodynamics and bowel viability have stabilized, cautious enteral nutrition is generally preferred because it supports mucosal integrity [11,37]. Parenteral nutrition is appropriate when prolonged enteral intolerance or major bowel resection is anticipated; critical-care nutrition guidance generally supports initiation within several days, when enteral feeding remains contraindicated [37]. Patients with extensive resection require early specialist nutritional assessment. The functional consequences depend not only on remaining small-bowel length but also on preservation of the ileocecal valve and colon [37,38]. Some patients initially dependent on parenteral nutrition achieve intestinal adaptation and can later be weaned [38].

Outcomes

Mortality is driven by diagnostic delay, extent of bowel necrosis, severity of the underlying illness, persistent shock, multi-organ failure, advanced age, acidosis, high lactate, and escalating vasopressor requirements [7,9,16,28,30]. Extensive mesenteric atherosclerosis may reduce perfusion reserve and has been associated with worse outcomes, even when no acute occlusion is identified [39].

A recent study emphasized that conventional survival statistics underestimate the long-term burden. Many survivors remain dependent on institutional care, rehabilitation, dialysis, nutritional support, or assistance with daily activities, and only a minority regain their previous functional status [40]. This has implications for shared decision-making, particularly in patients with profound pre-existing frailty, irreversible injuries, or multi-organ failure.

Nevertheless, poor aggregate outcomes should not produce therapeutic nihilism. NOMI is potentially reversible when recognized before transmural infarction. Patients with early vasoconstriction, correctable hemodynamic triggers, and a favorable response to resuscitation or vasodilator treatment may recover without major resection [5,6,11].

Proposed practical management algorithm

The management of NOMI should follow a structured, stepwise approach, aimed at restoring mesenteric perfusion, while rapidly identifying patients who have already developed irreversible bowel injury [6,11]. The first step is early recognition. NOMI should be suspected in critically ill patients with cardiogenic or septic shock, severe heart failure, recent cardiac or aortic surgery, major hemorrhage, renal replacement therapy, or escalating vasopressor requirements, who develop unexplained abdominal pain, abdominal distension, ileus, metabolic acidosis, rising lactate levels, gastro-intestinal bleeding, or otherwise unexplained clinical deterioration.

Once NOMI is suspected, hemodynamic stabilization should begin immediately, without waiting for definitive imaging. Management should focus on correcting hypovolemia, optimizing cardiac output and oxygen delivery, treating the underlying cause of shock, correcting anemia and electrolyte disturbances, and reducing vasopressor doses to the lowest effective level, whenever clinically feasible. Broad-spectrum antibiotics should be initiated because disruption of the intestinal mucosal barrier may rapidly lead to bacterial translocation and sepsis.

After initial stabilization, urgent multiphase CTA should be performed to exclude occlusive AMI and evaluate for findings suggestive of NOMI, including reduced bowel-wall enhancement, bowel-wall thickening, pneumatosis intestinalis, portal venous gas, or bowel perforation. Simultaneously, patients at risk for IAH should undergo measurement of intra-abdominal pressure, as ACS requires prompt recognition.

The next step is assessment for bowel infarction. Patients with generalized peritonitis, perforation, uncontrolled sepsis, or radiological evidence strongly suggestive of transmural necrosis should proceed directly to emergency laparotomy, without delaying treatment for angiography or endovascular intervention. During surgery, damage-control principles should be adopted, whenever appropriate, with resection of clearly non-viable bowel, preservation of questionable segments, temporary abdominal closure, and routine consideration of a planned second-look laparotomy within 24-48 hours.

Conversely, patients without evidence of irreversible bowel necrosis should initially continue optimized medical management, including correction of the underlying low-flow state, hemodynamic optimization, and close clinical reassessment. In patients with persistent clinical suspicion of NOMI despite initial resuscitation, particularly when mesenteric vasospasm is strongly suspected, and appropriate expertise is available, selective mesenteric angiography with catheter-directed vasodilator therapy should be considered. Continuous intra-arterial infusion of papaverine is the most commonly employed strategy, although prostaglandin E1 may also be used. During treatment, careful hemodynamic monitoring is essential because systemic hypotension may occur, and anticoagulation may be considered in the absence of contraindications.

Finally, ongoing reassessment is mandatory, as NOMI is a dynamic disease. Serial clinical examinations, hemodynamic monitoring, lactate trends, acid-base status, organ function, and repeat imaging, when indicated, should guide further management. Persistent deterioration despite optimal medical or endovascular therapy should prompt immediate surgical exploration. Following recovery, nutritional support should be introduced cautiously, beginning with delayed enteral feeding, once intestinal perfusion has stabilized, while parenteral nutrition should be used when prolonged bowel dysfunction or extensive intestinal resection precludes adequate enteral intake.

**Figure 1:**
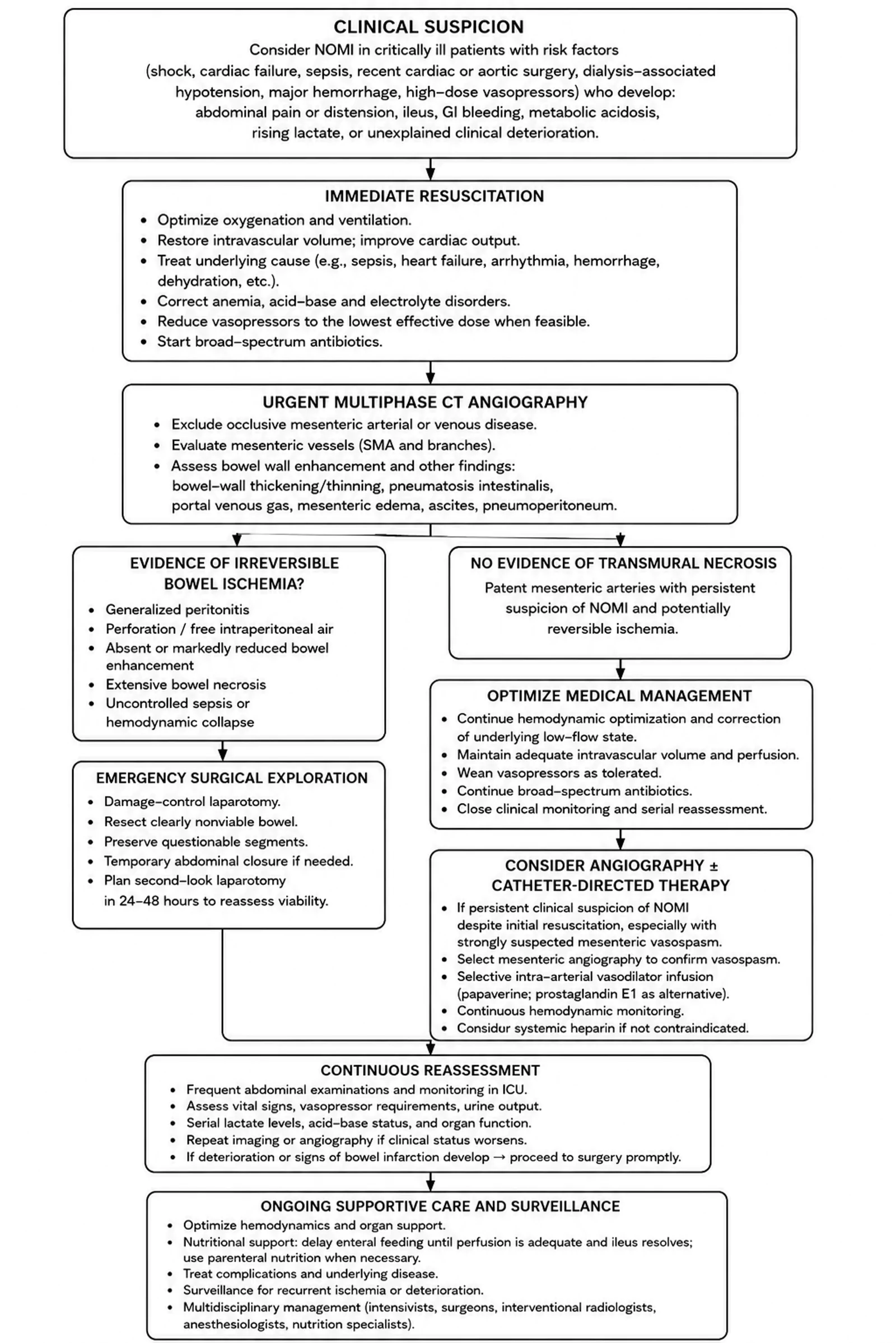
Proposed practical algorithm for the management of non-occlusive mesenteric ischemia CT: computed tomography; GI: gastrointestinal; ICU: intensive care unit; NOMI: non-occlusive mesenteric ischemia; SMA: superior mesenteric artery

Overall, successful treatment depends on rapid recognition, immediate correction of the underlying circulatory disorder, early CTA, timely selection of patients for catheter-directed vasodilator therapy, prompt surgery for irreversible bowel ischemia, and continuous multidisciplinary reassessment throughout the patient's clinical course [6,11]. The contemporary diagnostic and therapeutic interventions in NOMI are summarized in Table 1, while the proposed practical management algorithm is presented in Figure 1.

**Table 1:** Summary of the diagnostic and therapeutic interventions in non-occlusive mesenteric ischemia ACS: abdominal compartment syndrome; AMI: acute mesenteric ischemia; CTA: computed tomography angiography; NOMI: non-occlusive mesenteric ischemia; RCT: randomized controlled trial

Intervention	Evidence	Main findings	Major limitations	Current role
Hemodynamic optimization	Physiological studies, observational cohorts, guidelines	Fundamental treatment: correction of the low-flow state improves mesenteric perfusion	No NOMI-specific comparative trials	Cornerstone of management
Broad-spectrum antibiotics	Guidelines, observational studies	May reduce bacterial translocation and septic complications	Limited direct evidence	Recommended in suspected bowel ischemia
Vasopressor optimization	Critical care literature	Lowest effective dose recommended; no proven mesenteric-sparing vasopressor	Indirect evidence	Individualized management
CTA	Observational studies and guidelines	First-line imaging modality; excludes occlusive AMI and detects bowel injury	Early disease may have subtle findings	First-line diagnostic test
Catheter angiography	Observational studies	Diagnostic confirmation and therapeutic access	Invasive; limited availability	Selected patients
Intra-arterial papaverine	Systematic review and observational studies	Associated with improved mesenteric perfusion and lower mortality	Selection bias; no RCTs	Consider in potentially reversible NOMI
Surgery	Surgical series and guidelines	Mandatory for transmural necrosis, perforation, or peritonitis	Timing difficult	Definitive treatment for irreversible ischemia
Second-look laparotomy	Surgical series	Preserves viable bowel and detects evolving ischemia	No comparative trials	Recommended when bowel viability is uncertain
Decompression for ACS	Systematic review and guidelines	Improves organ perfusion and reduces intra-abdominal pressure	High mortality reflects disease severity	Mandatory when ACS develops

Evidence limitations and future directions

The available evidence is limited by inconsistent diagnostic definitions, the predominantly retrospective and single-center design of the published studies, small patient cohorts, and substantial methodological heterogeneity. Diagnostic criteria for NOMI vary considerably, with studies relying on angiographic findings, operative findings, endoscopy, CTA, or clinical diagnosis, while the distinction between reversible hypoperfusion, mucosal ischemia, and transmural necrosis is not standardized. Similarly, indications for catheter-directed therapy, timing of intervention, vasodilator regimens, surgical strategies, and outcome definitions differ substantially across studies. Many cohorts also span prolonged study periods during which diagnostic techniques, critical care management, imaging protocols, and endovascular expertise evolved considerably. Furthermore, treatment groups are inherently subject to selection bias, as patients undergoing angiography must survive long enough and be sufficiently stable for intervention, whereas those managed conservatively may represent patients considered too unstable or beyond salvage. Consequently, direct comparison across studies is difficult, and the reported outcomes should be interpreted cautiously, because they are susceptible to selection bias, survivor bias, and confounding by indication.

Randomized trials of intra-arterial vasodilators are lacking. Future studies should define standardized eligibility criteria, dosing regimens, treatment-response endpoints, catheter-management protocols, and triggers for surgery. Comparative studies should measure not only mortality but also bowel-resection length, intestinal failure, intensive-care duration, major complications, functional recovery, and quality of life.

Automated analysis of longitudinal clinical data may improve recognition. Potential models could integrate vasopressor dose, lactate kinetics, arterial pressure variability, cardiac output, feeding intolerance, intra-abdominal pressure, laboratory markers, and quantitative CTA features. Artificial intelligence (AI) has been proposed as a means of detecting subtle combinations of physiological and radiological abnormalities, but prospective external validation and clinically interpretable thresholds are required before deployment. Other priorities include quantitative perfusion CTA, dual-energy CTA, standardized indocyanine-green fluorescence analysis, bedside microcirculatory monitoring, and validated biomarkers of enterocyte injury. Multicenter registries using uniform definitions are needed because the rarity and heterogeneity of NOMI make conventional trials difficult.

Discussion

NOMI remains one of the most challenging conditions encountered in critical care. Despite substantial advances in intensive care medicine, CTA, and endovascular techniques, mortality continues to exceed 60% in most contemporary series, reflecting the combination of delayed diagnosis, advanced underlying illness, and rapid progression to irreversible intestinal necrosis [7,9,11]. Unlike embolic or thrombotic AMI, NOMI is fundamentally a disorder of systemic hypoperfusion and microvascular dysfunction, rather than large-vessel occlusion [3-6]. Consequently, successful treatment requires simultaneous management of the underlying circulatory disturbance and the intestinal consequences of prolonged hypoperfusion.

A recurring theme throughout the literature is the absence of high-quality evidence supporting many current therapeutic recommendations [6,11,27]. Most available data originate from retrospective single-center studies, small observational cohorts, or expert consensus, with considerable heterogeneity in diagnostic criteria, patient selection, therapeutic protocols, and reported outcomes [27,28,30]. This variability makes direct comparison between studies difficult and limits the strength of any recommendations. As a result, most recommendations regarding catheter-directed vasodilator therapy, anticoagulation, nutritional support, and even operative timing remain based on low- or very low-quality evidence.

Perhaps the greatest controversy concerns the role of catheter-directed intra-arterial vasodilator therapy. Physiologically, selective infusion of papaverine or prostaglandin E1 is an attractive strategy because it directly targets the mesenteric vasoconstriction that characterizes NOMI. Several observational studies suggest improved survival compared with conventional treatment alone [3,5,27,28,30]. However, these findings should be interpreted cautiously, because patients selected for angiography are often diagnosed earlier, are more hemodynamically stable, and may represent a subgroup with potentially reversible disease. Whether the apparent benefit results from the pharmacological intervention itself or from earlier recognition, multidisciplinary management, and selection bias remains uncertain. Randomized controlled trials are unlikely because of the rarity of NOMI and the ethical challenges of withholding potentially life-saving therapy in critically ill patients. Consequently, well-designed prospective multicenter registries may represent the most realistic approach to strengthening the evidence base.

Another important observation is that management should not focus exclusively on restoring mesenteric blood flow. NOMI frequently develops as part of a broader syndrome of circulatory failure, emphasizing that optimization of systemic hemodynamics, treatment of sepsis or cardiogenic shock, correction of hypovolemia, minimization of vasopressor exposure, and early recognition of ACS are equally important therapeutic priorities [6,11]. Similarly, surgery should not be regarded as a treatment failure, but rather as an integral component of management once irreversible bowel injury has occurred. Adoption of damage-control principles, preservation of borderline bowel segments, and routine consideration of second-look laparotomy acknowledge the dynamic and often reversible nature of intestinal ischemia during the early stages of the disease [5,6,11].

The diagnostic pathway also deserves critical consideration. Although multiphase CTA has largely replaced catheter angiography as the initial imaging modality, radiological findings remain imperfect, particularly during the earliest phases of ischemia [11,22-26]. Likewise, no currently available biomarker possesses sufficient sensitivity or specificity to reliably establish or exclude the diagnosis [18,19]. This reinforces the importance of maintaining a high index of clinical suspicion, particularly in critically ill patients, whose abdominal findings may be subtle or completely absent because of sedation or mechanical ventilation [5,6]. Future progress will likely depend on combining serial physiological variables, laboratory biomarkers, quantitative imaging parameters, and AI-assisted prediction models, rather than relying on any single diagnostic test.

Overall, the available evidence supports a multidisciplinary, time-dependent treatment strategy, centered on rapid recognition, aggressive correction of the underlying low-flow state, early CTA, selective use of catheter-directed vasodilator therapy in carefully selected patients, prompt surgical intervention for irreversible bowel ischemia, and meticulous postoperative critical care. Although important uncertainties remain, earlier diagnosis and standardized institutional management pathways probably represent the interventions most likely to improve outcomes in this devastating disease.

## Conclusions

NOMI remains one of the most lethal abdominal emergencies because diagnosis is frequently delayed, and patients often present with advanced systemic illness. Early recognition in high-risk patients, prompt correction of the underlying low-flow state, and urgent multiphase CTA are the cornerstones of management. Catheter-directed intra-arterial vasodilator therapy may benefit carefully selected patients without established bowel infarction, whereas immediate surgery is mandatory when irreversible intestinal ischemia is suspected. Given the limited quality of the available evidence, management should follow a multidisciplinary, individualized approach. Future prospective multicenter studies are needed to refine patient selection, standardize treatment strategies, and improve the persistently poor outcomes associated with this critical condition.
